# Construction of efficient *Streptococcus zooepidemicus* strains for hyaluoronic acid production based on identification of key genes involved in sucrose metabolism

**DOI:** 10.1186/s13568-016-0296-7

**Published:** 2016-11-28

**Authors:** Xuzhen Zhang, Man Wang, Tuanjie Li, Lixia Fu, Wei Cao, Hao Liu

**Affiliations:** MOE Key Lab of Industrial Fermentation Microbiology, College of Biotechnology, Tianjin University of Science & Technology, Tianjin, 300457 China

**Keywords:** Hyaluoronic acid, Metabolic engineering, *Streptococcus zooepidemicus*

## Abstract

Biosynthesis of polysaccharide hyaluoronic acid (HA) by *Streptococcus zooepidemicus* is a carbon-intensive process. The carbon flux and factor(s) restricting HA yield were not well understood. Here, we investigated the function of genes involved in sucrose metabolism and identified targets limiting HA yield, which were exploited to construct efficient *S. zooepidemicus* strains for HA production. The sucrose uptake was addressed by deletion of *scrA* and *scrB*, which encodes sucrose-PTS permease and sucrose-6-phosphate hydrolase, respectively. We found that *scrB* was essential for the growth of *S. zooepidemicus* and HA biosynthesis, and accumulation of sucrose-6-phosphate was toxic. Δ*scrB* could not grow in THY-sucrose medium, while Δ*scrA* and Δ*scrA*Δ*scrB* showed negligible growth defects. Overexpression of *scrA* significantly reduced biomass and HA production, while overexpression of *scrB* resulted in 26% increase of biomass and 30% increase of HA yield. We revealed that fructose-6-phosphate for HA biosynthesis mainly originates from glucose-6-phosphate. Deletion of *scrK,* a gene encoding hexokinase, led to 11% reduction of biomass and 12% decrease of HA yield, while deletion of *hasE*, a gene encoding phosphoglucoisomerase, resulted in the abolishment of HA biosynthesis and a significantly slow growth. We found that HA biosynthesis could be improved by directing carbon flux to fructose-6-phosphate. Deletion of *fruA* encoding the EII of fructose-PTS and *fruK* encoding phosphofructokinase showed no apparent effect on cell growth, but resulted in 22 and 27% increase of HA yield, respectively. Finally, a strain with 55% increase of HA was constructed by overexpression of *scrB* in Δ*fruK*. These results provide a solid foundation for further metabolic engineering of *S. zooepidemicus* for highly efficient HA production.

**Electronic supplementary material:**

The online version of this article (doi:10.1186/s13568-016-0296-7) contains supplementary material, which is available to authorized users.

## Introduction

Hyaluronic acid (HA) is a linear polysaccharide consisting of 2000–25,000 repeating disaccharide units of d-glucuronic acid (GlcUA) and N-acetylglucosamine (GlcNAc) linking alternatively by β-1, 3 and β-1,4 glycosidic bonds (Chong et al. 2005). The high molar mass and unique viscoelastic and rheological properties render this natural biopolymer a broad range of biomedical and industrial applications (Kogan et al. 2007). HA is found in connective tissues of animals as well as in the capsules of various bacteria such as *Streptococci* and *Pasteurella* (Wessels et al. 1991). Conventionally HA was extracted from animal tissues like rooster combs, and now is increasingly produced by fermentation of *Streptococcus zooepidemicus* owing to the simple purification process and low production cost (Liu et al. 2008a, b; Chen et al. 2009a, b).

Microbial synthesis of HA is a carbon- and energy-intensive process (Chong and Nielsen 2003; Chong et al. 2005; Ruffing and Chen 2006). The synthesis of HA accounts for about 5% carbon source, while cell growth and production of lactic acid and acetic acid consume around 10% and 80% carbon source, respectively (Liu et al. 2008a, b). Precursors, such as uridine diphosphate-glucuronic acid and uridine diphosphate-N-acetyl glucosamine, for HA synthesis are also precursors for cell wall biosynthesis. Therefore, HA synthesis competes with the cell growth for carbon source and energy. It is reasonably expected that high yield of HA can be achieved by decreasing the competition of cell growth and inhibition effect of lactic acid on synthesis. Thus, optimization of nutrition and culture condition and use of various fermentation modes have been attempted to enhance HA yield in *S. zooepidemicus* (Liu et al. 2008a, b; Pires and Santana 2010).

The metabolic engineering approach has been explored to increase HA yield and control HA molecular weight in *S. zooepidemicus*. Overexpression of NADH oxidase resulted in 33% and 15% increase of ATP and biomass, respectively, but no improvement for HA yield was observed in *S. zooepidemicus* (Chong et al. 2005). Optimization of HA precursor levels using feeding or genetic engineering approaches can improve HA molecular weight (Chen et al. 2009a, b, 2014). Moreover, recombinant HA production has been exploited in various bacteria and yeast (Widner et al. 2005; Mao and Chen 2007; Yu and Stephanopoulos 2008; Liu et al. 2011; Jeong et al. 2014). Owing to the limited knowledge of gene function and physiology of *S. zooepidemicus*, few cases of desired increase of HA yield were reported using metabolic engineering strategy. Release of complete genome sequences of several *S. zooepidemicus* strains and successful development of a markerless gene-deletion system enable us to elucidate the role of individual genes in cell growth and metabolism, which will guide the metabolic engineering of *S. zooepidemicus* for HA production (Beres et al. 2008; Ma et al. 2011; Sun et al. 2013).

To identify target(s) for metabolic engineering of *S. zooepidemicus*, we extended the previous study of the HA biosynthesis pathway by systematically investigating the function of genes involved in sucrose uptake and metabolism. We found that *scrB* was essential for the growth and HA production in the presence of sucrose. Overexpression of *scrB* resulted in 15% increase of biomass and 23% increase of HA yield. *fruA* and *fruK* play important roles in the control of carbon flux to HA biosynthesis. Deletion of *fruA* or *fruK* resulted in 22% and 27% increase of HA yield respectively. Up to 55% increase of HA yield was achieved by overexpressing *srcB* in Δ*fruK* mutant cells.

## Materials and methods

### Bacterial strains and growth conditions

All strains used in this study are listed in Additional file 1: Table S1. *Streptococcus. equi* subsp*. zooepidemicus* ATCC39920 (*S. zooepidemicus*) wild type (WT) and mutants were grown at 30 °C or 37 °C in Todd-Hewitt yeast (THY) medium (Sun et al. 2013) or chemically defined medium II (CDM2) (Armstrong and Johns 1997) *Escherichia coli* (*E. coli*) JM109 was grown at 37 °C in Luria–Bertani (LB) medium supplemented with antibiotics when necessary (Liu et al. 2007). The concentrations of antibiotics used in experiments were as follows: for *E. coli*, ampicillin (100 μg/mL), and spectinomycin (50 μg/mL), and for *S. zooepidemicus*, spectinomycin (100 μg/mL).

### Gene deletion in *S. zooepidemicus*

Genes were deleted using a markerless gene-deletion system as described previously (Sun et al. 2013). Briefly, using *S. zooepidemicus* genomic DNA as the template, the upstream and downstream fragments of *scrA* were amplified by PCR and joined by splicing overextension (SOE) PCR. The PCR products were separated by 1% agarose gel electrophoresis, and subsequently excised from the gel and purified with Gel extraction Kit (Qiagen, Hilden, Germany). The resultant product was digested and ligated into the *Sal*I/*Eco*RI sites of the vector pSET4s::sacB to obtain pSET4s::sacB::scrALR. *S. zooepidemicus* containing pSET4s::sacB::scrALR was first grown at 30 °C for 12 h and then further cultured at 37 °C for another 4 h in THY medium supplemented with 100 μg/mL spectinomycin. The culture was selected on THY medium supplemented with 5% (w/v) sucrose. The sucrose-resistant and spectinomycin-sensitive clones were isolated, and *scrA* gene-deletion mutants were examined by PCR and further confirmed by sequencing. The same strategy as used for *scrA* deletion was followed to construct other single-gene-deficient strains and double mutants. The primers used for construction of gene deletion cassettes and selection of mutants are listed in Additional file 1: Table S2. The restriction enzyme sites are underlined.

### Generation of *scrA* or *scrB* overexpression strains

Genomic DNA of *S. zooepidemicus* was used as the template for cloning of *scrA* and *scrB*. In brief, the open reading frame (ORF) of *scrA* or *scrB* together with its 200 bp promoter region was amplified by PCR. After purification, the resultant products were digested and then ligated onto plasmid pLH243, a modified pSET4S vector, to obtain pLH243::scrA and pLH243::scrB, respectively. The fidelity of cloned sequence was confirmed by sequencing. pLH243::scrA or pLH243::scrB was introduced into wild-type *S. zooepidemicus*, Δ*fruA* or Δ*fruK* and then selected with spectinomycin to obtain transformants that express extra copy of *scrA* or *scrB* contained on the plasmid. The primers used for construction of *scrA* or *scrB* overexpression cassette are listed in Additional file 1: Table S2. The restriction enzyme sites are underlined.

### Fermentation

Batch fermentation of *S. zooepidemicus* wild type and mutants was carried out in a 5-L bioreactor (Sartorius Stedim, Aubagne, France) with a working volume of 3 L as described (Chen et al. 2009a, b). The fermentation medium is composed of (per liter) 50 g sucrose, 3.5 g yeast extract, 10 g casein peptone, 2 g K_2_HPO_4_, 1.5 g NaCl, and 0.4 g MgSO_4_·7H_2_O. During fermentation process, the pH was maintained at 7.0 by automatic addition of 5 M NaOH, and temperature was controlled at 37 °C with agitation at a speed of 400 rpm and aeration volume 1.5 vvm. Flask experiments were conducted using 250-mL conical flasks (100 mL culture volume) containing sucrose -THY (in g/L: beef extract 10, casein tryptone 20, sucrose 2, yeast extract 2, NaHCO_3_ 2, NaCl 2, Na_2_HPO_4_ 0.4) with agitation (200 rpm) at 37 °C. The pH was initially set to 7.0 and adjusted every 2–3 h with sterile 5 M NaOH.

### Analytic methods

HA concentration was determined by the carbazole methods described previously (Bitter and Muir 1962), where the optical density (OD) was measured at 530 nm using a spectrophotometer (UV-2100 spectrophotometer). Cell concentration was determined by measuring the OD of the culture at 660 nm. The concentration of lactic acid was determined by Biosensing meter (SBA-40E). Sucrose concentration was determined by resorcinol method (Liu et al. 2008a, b). In brief, 0.9 mL sucrose sample mixed with 0.1 mL 2 M NaOH was incubated in boiled water for 10 min and then immediately cooled in running water. 1 mL 10 M resorcinol and 3 mL 10 M HCl were sequentially added into the mixture followed by incubation in 80 °C water for 8 min and then cooled to room temperature. The absorbance was measured at 500  nm and the sucrose concentration was determined by the standard curve.

## Results

### *scrB* is essential for the growth of *S. zooepidemicus* on sucrose-containing media

**Fig. 1:**
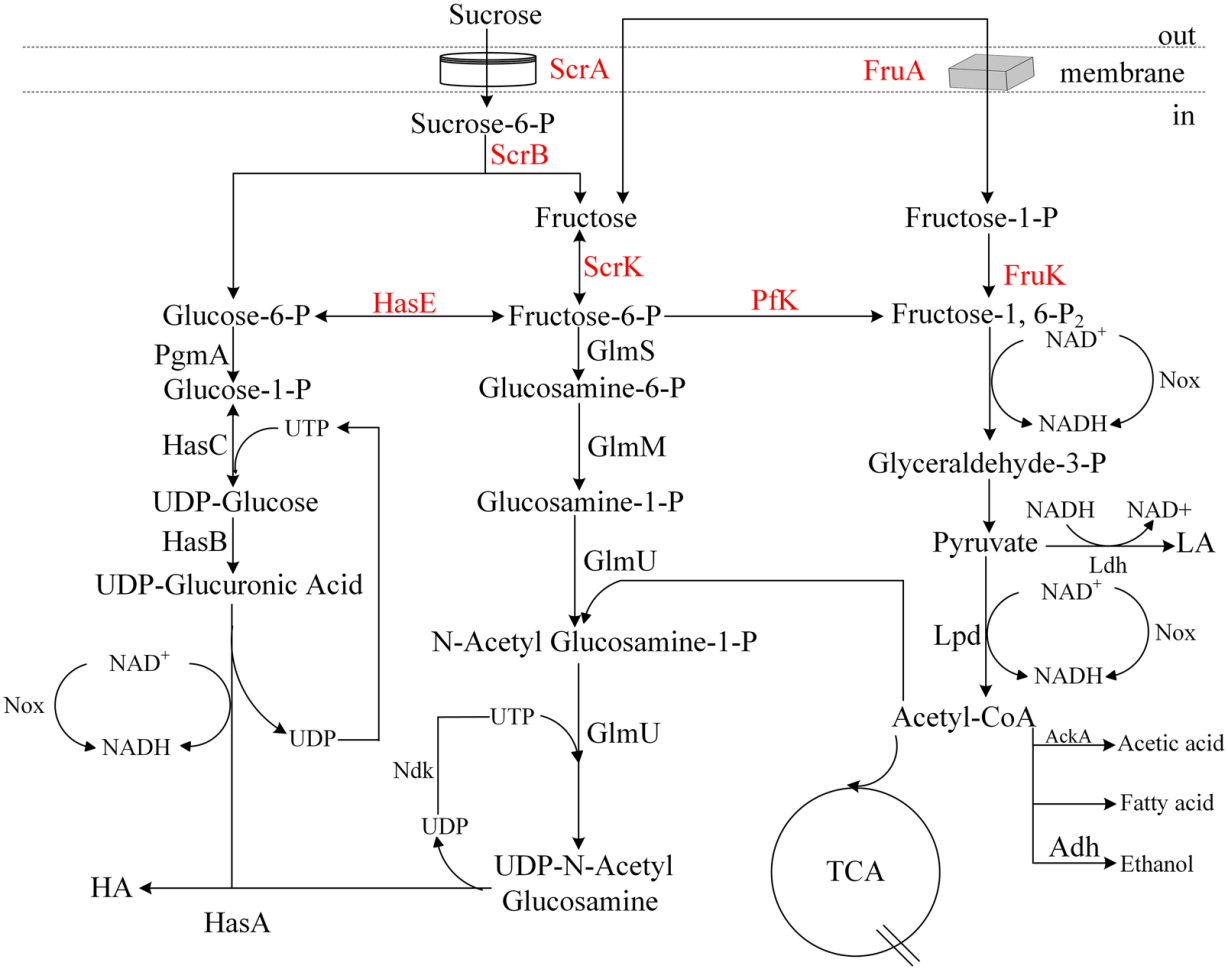
The predicted sucrose metabolic and HA biosynthetic pathways. The *arrow* indicates the direction of carbon flux

**Fig. 2:**
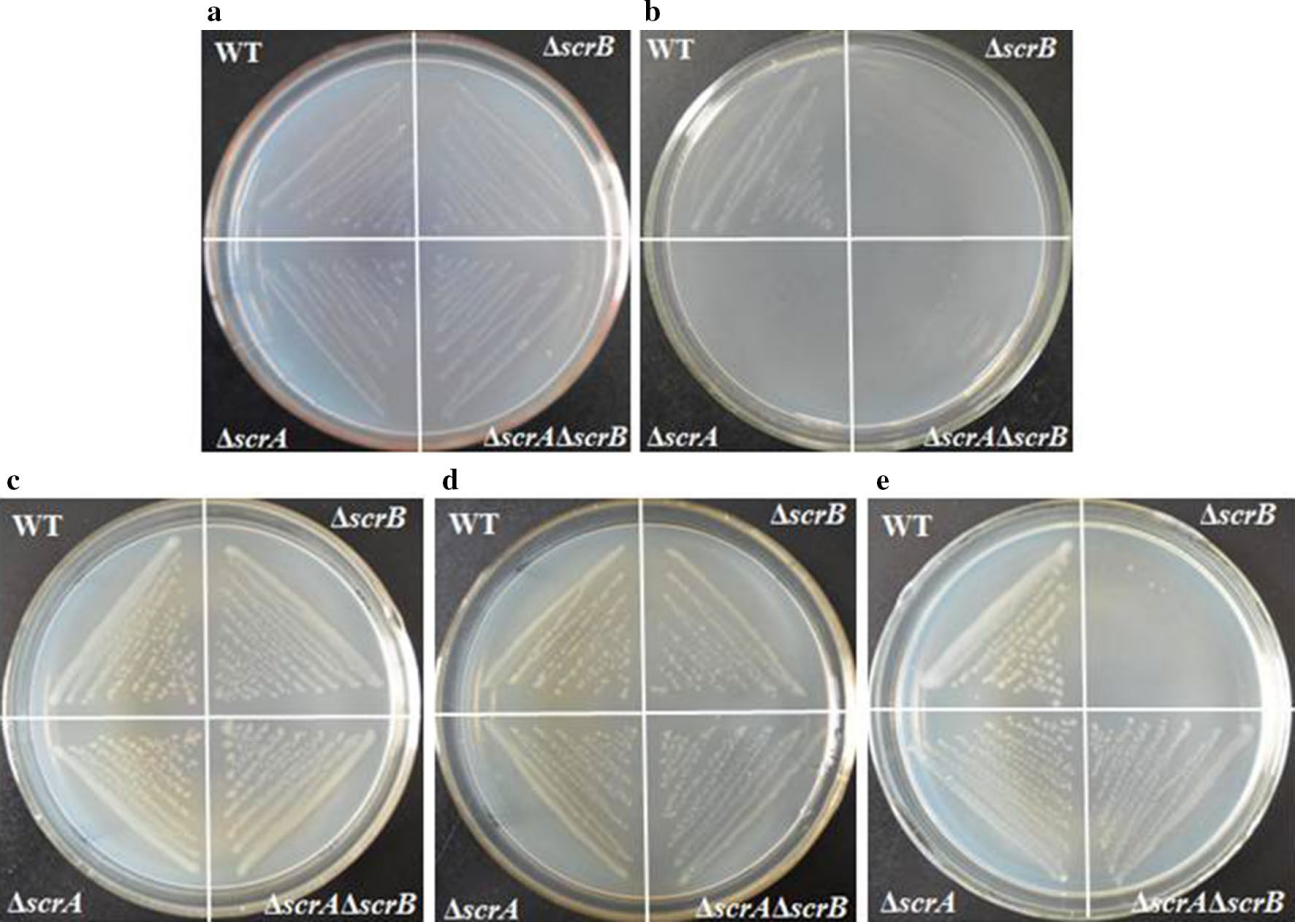
Growth profiles of *srcA*- or/and *scrB*-deficient strains. Wild type (WT) and the indicated mutants were grown on **a** CDM2 (glucose+), **b** CDM2 (sucrose+), **c** THY (glucose+), **d** THY (glucose−), **e** THY (sucrose+) for 24 h and the colonies were photographed. Carbon in the medium is showed in the *brackets*. + means inclusion of the sugar, − means exclusion of the sugar

*S. zooepidemicus* can ferment sucrose to HA, and the pathway of sucrose metabolism is shown in Fig. 1. *S. zooepidemicus* likely depends on the PEP-dependent phosphotransferase system (PTS) for sucrose transportation as well as its phosphorylation. Genome-wide analysis suggests that *S. zooepidemicus* contains a single copy of *scrA* and *scrB*, which are predicted to encode the Enzyme II of sucrose-PTS and sucrose-6-phosphotate hydrolase, respectively. Hypothetically, ScrA transports environmental sucrose into the cell and phosphoralates it to form sucrose-6-phosphotate, while ScrB hydrolyzes sucrose-6-phosphotate to glucose-6-phosphate and fructose (Reid and Abratt 2005), which are subsequently utilized by the cell for growth and HA synthesis. To address the utilization of sucrose by *S. zooepidemicus*, we first deleted *scrA* and *scrB*, and constructed Δ*scrA* and Δ*scrB* single mutants and Δ*scrA*Δ*scrB* double mutant. These three mutants showed no significant differences from wild type when the strains were cultured on solid chemical defined medium II (CDM2), in which glucose was the sole carbon source (Fig. 2a). However, Δ*scrA*, Δ*scrB* and Δ*scrA*Δ*scrB* could not grow on the plate while glucose was replaced by sucrose in the medium (Fig. 2b). These observations suggest that *scrA* and *scrB* are indispensable for the utilization of sucrose by *S. zooepidemicus* although neither is essential for glucose metabolism.

We further performed growth assay on more complex media. All mutants grew as well as wild type on THY medium in which glucose was the main carbon source (Fig. 2c). Exclusion of glucose from THY medium did not make apparent differences to the growth of the mutants and wild type (Fig. 2d), suggesting that the minimal complex carbon source in THY medium is sufficient for the growth of these strains. Significantly, replacement of glucose with sucrose in THY medium resulted in growth inhibition of Δ*scrA*, Δ*scrA*Δ*scrB* and abolishment of growth of Δ*scrB* (Fig. 2e). In liquid sucrose-THY medium, Δ*scrA* and Δ*scrA*Δ*scrB* produced 45% less of biomass than wild type, and Δ*scrB* could not grow in this culture condition (data not shown). It is likely that Δ*scrA* and Δ*scrA*Δ*scrB* use the complex carbon source for growth even in the presence of high concentration of sucrose since these mutants were unable of transporting sucrose into cell. In contrast, Δ*scrB* is able to transport sucrose into the cell and form sucrose-6-phosphate, however, it is incapable of hydrolyzing sucrose-6-phosphate. Based on above data, we speculate that *scrB* is essential for the growth of *S. zooepidemicus* in the presence of sucrose, and high concentration of sucrose-6-phosphate is likely toxic for *S. zooepidemicus* and inhibits cell growth.

### Overexpression of *scrB* promotes *S. zooepidemicus* growth and HA biosynthesis

**Fig. 3:**
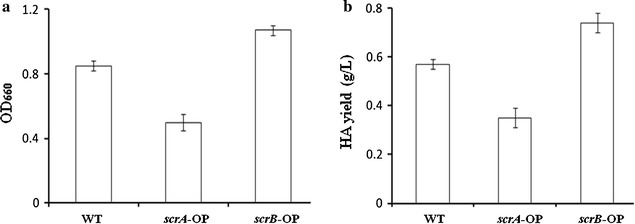
Effects of overexpression of *scrA* or *scrB* (*scrA*-OP or *scrB*-OP) on the growth and HA biosynthesis. **a** The indicated strains were grown in liquid THY (sucrose+) flasks for 24 h and cell density was determined by measuring the OD_660_. **b** HA produced by the indicated strains grown in liquid THY (sucrose+) flasks for 24 h was determined by the carbazole method. Data (**a**, **b**) represents the mean values from three independent experiments

It is common that accumulation of high level of toxic intermediate within cell inhibits its growth and productivity. To further confirm the observations on Δ*scrA* and Δ*scrB* and explore the possibility of increasing HA yield by modulation of sucrose-6-phosphate level, *scrA* and *scrB* were overexpressed in wild type, respectively. Analysis of the OD_660_ of cultures in liquid sucrose-THY showed that *scrA*-overexpression strain had about 41% less biomass than wild type, while *scrB*-overexpression strain produced around 26% more biomass than wild type (Fig. 3a). Compared with wild type, overexpression of *scrA* resulted in about 40% decrease of HA yield while overexpression of *scrB* led to around 30% increase of HA yield (Fig. 3b). These data suggests that high level of sucrose-6-phosphate restricts *S. zooepidemicus* growth and HA biosynthesis, while accelerating the hydrolysis of sucrose-6-phosphate by overexpression of *scrB* can promote cell growth and HA biosynthesis.

### Fructose-6-phosphate is mainly from glucose-6-phosphate

**Fig. 4:**
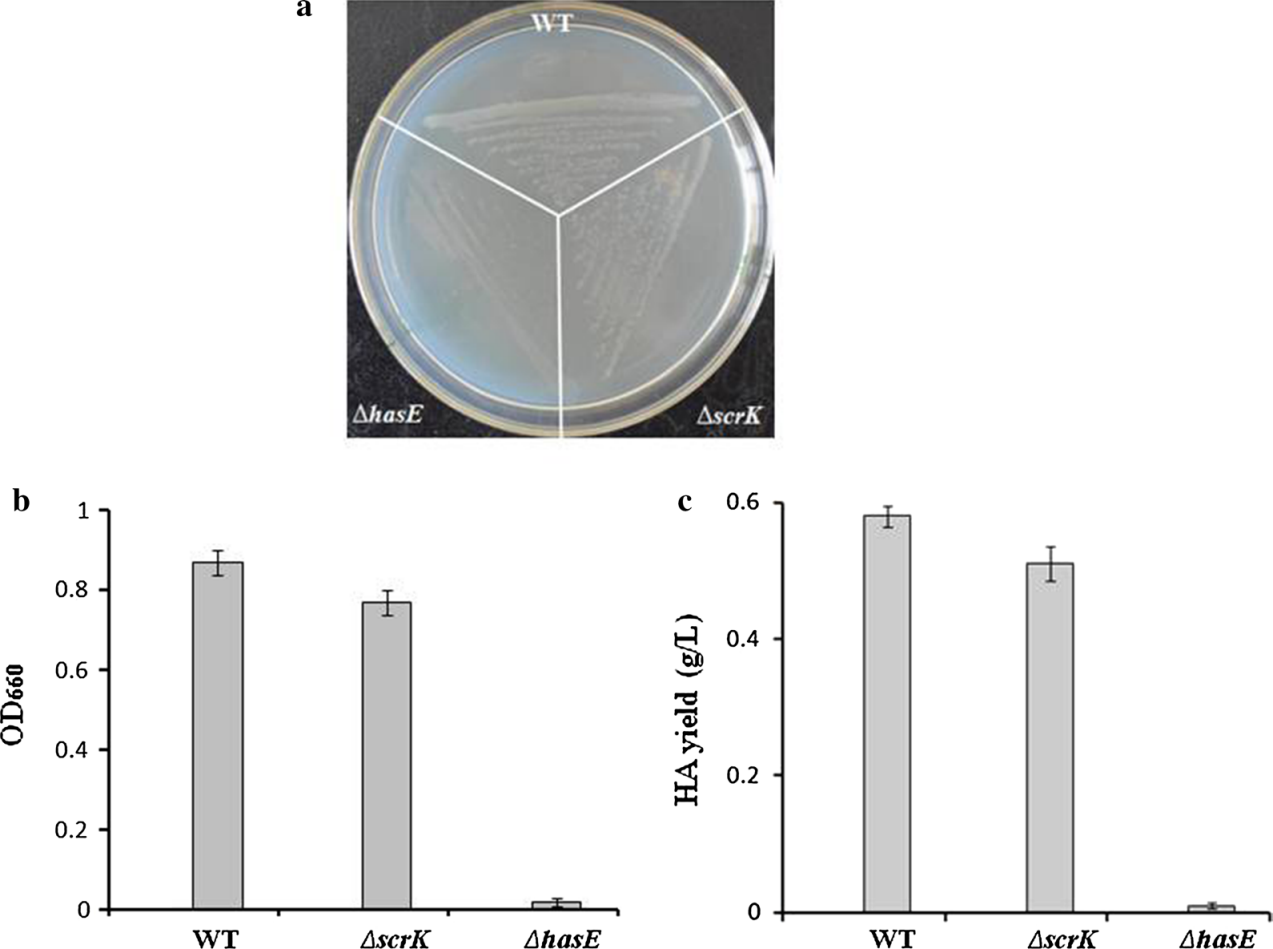
Effects of deletion of *hasE* or *scrK* on cell growth and HA biosynthesis. **a** WT and the indicated mutants were grown on solid THY (sucrose+) plates for 24 h, and the colonies were photographed. **b** The indicated strains were grown in liquid THY (sucrose+) flasks for 24 h, and the cell density was determined by measuring the OD_660_. **c** HA produced by the indicated strains grown in liquid THY (sucrose+) flasks for 24 h was determined by the carbazole method. Data (**b**, **c**) represents the mean values from three independent experiments

The d-glucuronic acid (GlcUA) and N-acetyl glucosamine (GlcNAc) moieties of HA are derived from glucose-6-phosphate and fructose-6-phosphate, respectively (Chong and Nielsen 2003). As depicted in Fig. 1, hydrolysis of sucrose-6-phosphate by ScrB produces glucose-6-phosphate and fructose. The genome of *S. zooepidemicus* contains a candidate gene *scrK*, probably encoding a fructokinase which converts fructose to fructose-6-phosphate. In an attempt to define the metabolic pathway of sucrose, we deleted *scrK* and investigated the phenotype of Δ*scrK*. Surprisingly, we found that loss of *scrK* showed moderate effects on cell growth and HA synthesis (Fig. 4a–c). Δ*scrK* had 89% biomass and 88% HA yield of wild type, suggesting that *scrK* plays a minor role in sucrose utilization. We showed previously that Δ*hasE*, a phosphoglucoisomerase deficient mutant, had significant growth defect in glucose-containing medium and could not ferment glucose to HA (Zhang et al. 2016). Here, a similar defect was observed with Δ*hasE* cultured in sucrose-containing media (Fig. 4a–c). Thus, we propose that when sucrose is the main carbon source, the function of *scrK* and *hasE* both contributes to intracellular fructose-6-phosphate level, while most of flucose-6-phosphate is converted from glucose-6-phosphate by HasE.

### Deletion of *fruA* or *fruK* increases HA yield

**Fig. 5:**
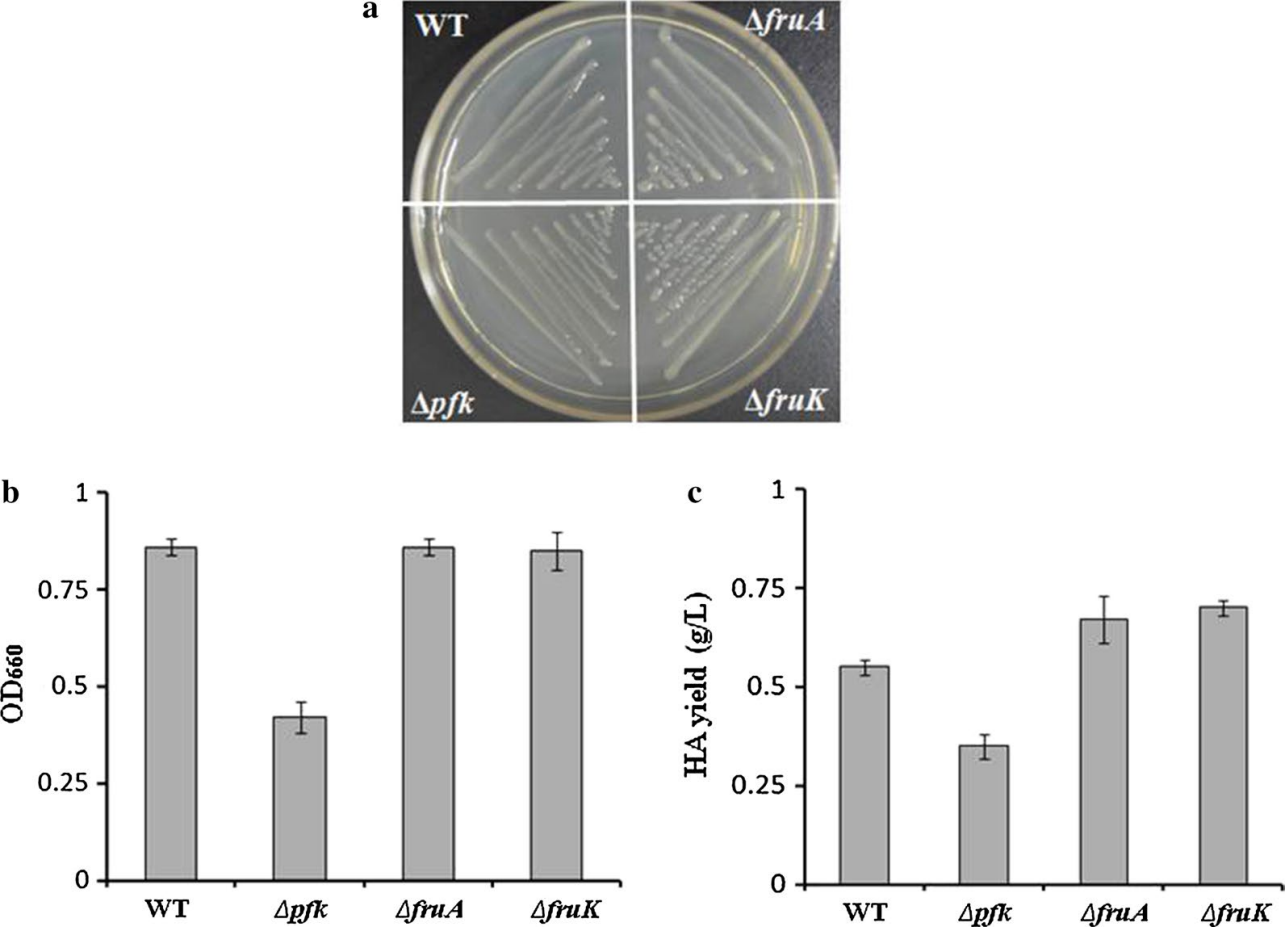
Effects of deletion of *hasE, fruA* or *fruK* on cell growth and HA biosynthesis. **a** WT and the indicated mutants were grown on solid THY (sucrose+) plates with for 24 h, and the colonies were photographed. **b** The indicated strains were grown in liquid THY (sucrose+) flasks for 24 h, and the cell density was determined by measuring the OD_660_. **c** HA produced by the indicated strains grown in liquid THY (sucrose+) flasks for 24 h was determined by the carbazole method. Data (**b**, **c**) represents the mean values from three independent experiments

When grown in liquid sucrose-THY, the culture of Δ*scrK* showed no significant differences from that of wild type in fructose levels (data not shown), suggesting that another pathway is involved in the metabolism of fructose. We found that the genome of *S. zooepidemicus* contains a fructose-PTS for the utilization of fructose, in which *fruA* and *fruK* encode the permease EII of fructose-PTS and phosphofructokinase, respectively. To address the physiological function(s) of *fruA* and *fruK* in sucrose metabolism, cell growth and HA biosynthesis, we deleted these two genes individually and characterized Δ*fruA* and Δ*fruK* strains. Phenotypic analysis of these mutants was performed by their culturing on solid and liquid sucrose-THY medium. Clone morphology and measurement of OD_660_ showed that Δ*fruA* and Δ*fruK* had no apparent differences from wild type (Fig. 5a, b). Interestingly, our analysis indicated that fructose levels of the cultures of Δ*fruA* and Δ*fruK* were comparable to that of wild type (data not shown). Thus, it is probably that the function of ScrK is enhanced in Δ*fruA* and Δ*fruK*, which favors the conversion of fructose to fructose-6-P. This change of metabolic flux likely promotes HA biosynthesis. Consistent with this hypothesis, a 22 and 27% increase of HA yield was observed with the flask cultures of Δ*fruA* and Δ*fruK*, respectively (Fig. 5c).

### Overexpression *of scrB* in Δ*fruA* or Δ*fruK* strain enhances HA production

**Fig. 6:**
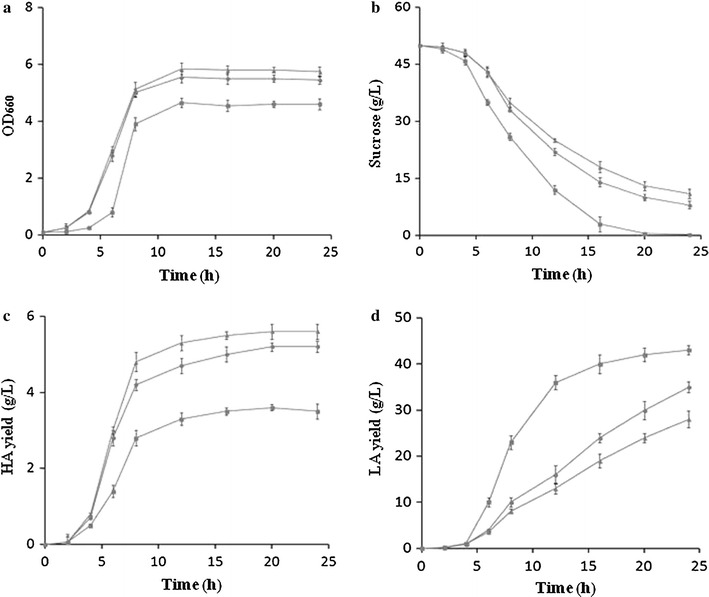
Fermentation analysis of WT, s*crB*/OP-Δ*fruA* and *scrB*/OP-Δ*fruK*. Batch fermentation of *S. zooepidemicus* wild type and mutants was carried out in a 5-L bioreactor as described in "Materials and methods" section. The samples taken at the indicated time point were analyzed. **a** Cell concentration was determined by measuring the OD_660_. **b** The concentration of lactic acid was determined by Biosensing meter. **c** The HA concentration was determined by the carbazole method. **d** Sucrose concentration was determined by resorcinol method. The symbols of *black square*, *black circle* and *filled triangle* represent WT, scrB/OP-Δ*fruA* and scrB/OP-Δ*fruK*, respectively. Data represents the mean values from three independent experiments

*scrB* was overexpressed in Δ*fruA* and Δ*fruK* to construct *scrB*/OP-Δ*fruA* and *scrB*/OP-Δ*fruK* strains, and fermentation assay was performed in 5 L fermentation tank to compare the cell growth, sucrose usage, HA yield and lactic acid production of these two engineered strains and wild type. As illustrated in Fig. 6a, *scrB*/OP-Δ*fruA* and *scrB*/OP-Δ*fruK* showed faster growth than wild type. Moreover, cell density (OD_660_) of *scrB*/OP-Δ*fruA* and *scrB*/OP-Δ*fruK* was 26 and 20% higher than that of wild type, respectively, in stationary phase (20 h). We found that *scrB*/OP-Δ*fruA* and *scrB*/OP-Δ*fruK* showed decreased ability to use sucrose. Around 10 and 13.6 g/L residual sucrose were detected in the 20 h fermentation broth of *scrB*/OP-Δ*fruA* and *scrB*/OP-Δ*fruK*, respectively, while sucrose was nearly depleted in the culture of wild type (Fig. 6b). In contrast, a significant increase of HA yield was observed with these two engineered strains. *scrB*/OP-Δ*fruA* and *scrB*/OP-Δ*fruK* produced 5.2 and 5.6 g/L HA, respectively, while wild type produced 3.6 g/L HA (Fig. 6c). Interestingly, an evident decrease of lactic acid production was observed with both *scrB*/OP-Δ*fruA* and *scrB*/OP-Δ*fruK* (Fig. 6d). These results demonstrate that accelerating the hydrolysis of sucrose-6-phosphate and manipulating the fructose metabolic pathway can efficiently direct the carbon flux to HA biosynthesis.

## Discussion

The role of the sucrose-specific PTS for sucrose metabolism has been studied in some detail (Reid and Abratt 2005). Our genetic characterization of *srcA* and *scrB* demonstrated that both of them are indispensible for the growth of *S. zooepidemicus* on CDM2 medium (Fig. 2b), in which sucrose is the sole carbon. Δ*scrB* grows well on complicated carbon source mixture glucose-THY medium, while it can not grow on sucrose-THY medium (Fig. 2c, e). The growth defect of Δ*scrB* can be complemented by plasmid-based expression of *scrB* complemented. It is likely that sucrose-6-phosphate accumulating intracellularly in Δ*scrB* as a consequence of uptake and phosphorylation of sucrose by ScrA is toxic for *S. zooepidemicus*. Similarly, growth of *Corynebacterium glutamicum* strains lacking sucrose-6-phosphate hydrolase was severely affected on a glucose–sucrose mixture (Engels et al. 2008). *Streptococcus mutans* mutant lacking sucrose-phosphate-hydrolyzing activity showed decreased growth in mannitol when sucrose was added to the culture medium (Zeng and Burne 2013). Thus, it could be a general phenomena that sucrose-6-phosphate is toxic for gram-positive bacteria.

Fructose 6-phosphate lies within the glycolysis metabolic pathway and is the substrate for the production of GlcNAc, the precursor of HA. Fructose 6-phosphate is produced by isomerisation of glucose-6-phosphate and phosphorylation of fructose by hexokinase or fructose kinase. *S. zooepidemicus* genome does not contain a gene encoding the putative hexokinase. Under sucrose environment, the deletion of *hasE* caused severe growth defects and the loss of HA production, while the deletion of *scrK* resulted in a marginal reduction in strain growth and HA production (Fig. 4a–c). The unexpected growth profile of Δ*hasE* and Δ*scrK* suggests that the function of *hasE* contributes most of the cellular fructose-6-phosphate level. *S. zooepidemicus* has two pathways for the metabolism of fructose, one is mediated by ScrK and the other is FruA and FruK. Deletion of *fruA* or *fruK* results in significant increase of HA production (Fig. 5c), suggesting that loss of either of these two genes likely promotes the carbon flux to HA biosynthesis. To further elucidate the underlying mechanism of *fruA* or *fruK* on HA biosynthesis, it will be necessary to investigate the expression profile of genes involved in HA biosynthetic pathway, the corresponding enzyme activity and the intermediate levels in *fruA*- and *fruK*-deficient strains.

Variant strategies, such increase of biomass and addition of intermediate chemicals, were explored to improve HA production in *S. zooepidemicus* (Chong et al. 2005; Liu et al. 2011). Alleviating the toxicity of metabolic intermediate promotes cell growth. Here, we found that overexpression of *scrB* significantly improves the growth and HA yield of *S. zooepidemicus* (Fig. 3b). Deletion of *fruA* or *fruK* likely increases fructose-6-phosphate level, resulting in increase of HA yield (Fig. 5c). Based on these finding, we constructed *scrB*/OP-Δ*fruA* and *scrB*/OP-Δ*fruK* strains, which showed significant increase in HA productivity (Fig. 6b). Compared with wild type, *scrB*/OP-Δ*fruA* and *scrB*/OP-Δ*fruK* produced less lactic acid, the side product, and had higher levels of residual sucrose (Fig. 6c, d). This suggests that both strains utilize sucrose more efficiently than wild type for HA biosynthesis. Recently, it is reported that down-regulation the expression of *pfkA*, a gene encoding phosphofructokinase, increase the HA yield in *Bacillus subtilis* (Jin et al. 2016). Here, we found that deletion of *pfk* in *S. zooepidemicus* results in inhibition of the growth and HA production (Fig. 5b, c). The distinct physiology of *B. subtilis* and *S. zooepidemicus* probably accounts for this difference.

In summary, our genetic investigation reveals that the function of *scrB* is essential for the growth of *S. zooepidemicus* and HA biosynthesis in the presence of sucrose. Characterization of Δ*hasE*,Δ*scrk*,Δ*fruA* and Δ*fruK* revealed the role of these genes in carbon flux and HA biosynthesis. Guided by these finding, a high efficient *scrB*/OP-Δ*fruK* was constructed, which showed 26% increase of biomass and 55% increase of HA yield.

## Additional file

**Additional file 1.** Additional tables.
