# Supplementary material for: Construction of efficient Streptococcus zooepidemicus strains for hyaluoronic acid production based on identification of key genes involved in sucrose metabolism
Source: AMB Express. 2016 Nov 28;6:121. doi: 10.1186/s13568-016-0296-7 (PMC5125315; doi:10.1186/s13568-016-0296-7)
Supplement: Supplementary file 1 — Additional file 1. Additional tables. [file 13568_2016_296_MOESM1_ESM.pdf]

Journal Name:

AMB Express

Manuscript Title:

Construction of Efficient *Streptococcus zooepidemicus* Strains for Hyaluronic Acid Production Based on Identification of Key Genes Involved in Sucrose Metabolism

Authors and Emails:

Xuzhen Zhang （15022010757@139.com）

Man Wang （13502069697@163.com）

Tuanjie Li （1163976479@qq.com）

Lixia Fu （fulixia321@163.com）

Wei Cao （caowei040108@tust.edu.cn）

Hao Liu* (liuhao@tust.edu.cn)

Authors' Affiliation:

MOE Key Laboratory of Industrial Fermentation Microbiology, College of Biotechnology, Tianjin University of Science & Technology, China

***Postal Address of Corresponding Author:**

NO.29, 13th Avenue, Tianjin Economic Development Area (TEDA)，Tianjin 300457, China

Table S1 Strains generated or used in this work

Strains	Locus	Protein	Source or reference
S. zooepidemicus ATCC39920 (Wild-type)			ATCC
E. coli JM109			Gibco BRL
Δ*scrA*	SeseC_00426	sucrose-phosphotransferase system (PTS), IIABC component	This work
Δ*scrB*	SeseC_00425	sucrose-6-phosphate hydrolase	This work
Δ*scrA*Δ*scrB*			This work
Δ*fruA*	SeseC_01452	fructose- phosphotransferase system (PTS), IIABC component	This work
Δ*fruK*	SeseC_01453	1-phosphofructokinase	This work
Δ*scrK*	SeseC_00430	fructokinase	This work
Δ*hasE*	SeseC_00235	glucose-6-phosphate isomerase	Zhang et al, 2016
Δ*pfk*	SeseC_01167	phosphofructokinase	
scrA/OP			This work
scrB/OP			This work
scrB/OP-Δ*fruA*			This work
scrB/OP-Δ*fruK*			This work

Table S2 Oligonucleotide primers used in this work

Primers	Sequence(5'-3')	Underlined sequence
Primers used for construction of the vectors for *scrA* gene deletion		
Primer81	ACGCGTCGACGTGATGTGGCCTGATTGGTC	*Sal*I
Primer82	AATCATCAACTCTTTTCAAGACCGATAGCATCTCCTTTTT	
Primer83	AAAAAGGAGATGCTATCGGTCTTGAAAAGAGTTGATGATT	
Primer84	CCGGAATTCTCATGCTTGGTTGCCAATTCC	*Eco*RI
Primers used for construction of the vectors for *scrB* gene deletion		
primer527	CGCGGATCCCACAATGAGCCACACTTCTG	*Bam*HI
primer528	CATCAAGCTCTAGTTCGGTGGGCAGAGCCAGAATAAGCGC	
primer529	GCGCTTATTCTGGCTCTGCCCACCGAACTAGAGCTTGATG	
primer530	TGGTCCATCCAGGCACCAATTAATTCGATGGGTTCCGAGG	
primer531	CCTCGGAACCCATCGAATTAATTGGTGCCTGGATGGACCA	
primer532	CCGGAATTCGCTGCTTGCTGTGCTTCAAC	*Eco*RI
Primers used for construction of the vectors for *fruA* gene deletion		
Primer41	CAACTGCAGCAGCGATTAGGCATTGATAA	*Pst*I
Primer42	ACGCGTCGACTCATCGAACCTCTACCTCAA	
Primer43	ACGCGTCGACTTATTGGCTGTAAAGCCAAACAT	
Primer44	CGCGGATCCGACTGAGGCATAGAGCTTGT	*Bam*HI
Primers used for construction of the vectors for *fruK* gene deletion		
Primer49	TAACTGCAGTTATCGTCAAGGCAATGTGG	*Pst*I
Primer50	ACGCGTCGACTCATAGTGCAATTACCTTCG	
Primer51	ACGCGTCGACATGAAAATACAGGATTTATT	
Primer52	CGCGGATCCGCATGGTAAACACTACCAGC	*Bam*HI
Primers used for construction of the vectors for *scrK* gene deletion		
Primer57	CGCGGATCCTGGATATGTCTCTCTTCCTG	*Bam*HI
Primer58	TATCAGATAAGCTCAAGTATCTTTTCTCCTTAGTAAATGA	
Primer59	TCATTTACTAAGGAGAAAAGATACTTGAGCTTATCTGATA	
Primer60	CCGGAATTCGCAGGTAGAGATCAAATGCT	*Eco*RI
Primers used for construction of the vectors for *hasE* gene deletion		
Primer331	CGGAATTCAGGCAGAGCAAGAGGTCAAG	*Eco*RI
Primer332	CATTACAAGCGTGCGTTGAGCGTTTCATTAGTATCATAACCT	
Primer333	AGGTTATGATACTAATGAAACGCTCAACGCACGCTTGTAATG	
Primer334	ACGCGTCGACCGTCCAACAGCCTGACTAAC	*Sal*I
Primers used for construction of the vectors for *pfk* gene deletion		
Primer33	CCGGAATTCAGGATTATTCAGCTGCTGAA	*Eco*RI
Primer34	ATTACCAGATCTTACTTATCTTTTTGGTACTCCTTTAATT	
Primer35	AATTAAAGGAGTACCAAAAAGATAAGTAAGATCTGGTAAT	
Primer36	CGCGGATCCGCAAATAATTGAACATGCTC	*Bam*HI
Primers used for examining double crossover events of *scrA* gene		
Primer85	AGTTGGTGACATCATTGTTG	
Primer88	ATGGAGCGTTCTATCCCTTC	
Primers used for examining double crossover events of *scrB* gene		

Primer77	CTCCTTGATCGGCTGCTTGAT	
Primer80	GGCTTAACATCACCTCCTGA	
Primers used for examining double crossover events of *fruA* gene		
Primer45	GCGAAGGTAATTGCACTATG	
Primer48	GTTGCCTTGGCCATCATCTT	
Primers used for examining double crossover events of *fruK* gene		
Primer53	AGACTATTGTGTTTCAGGCT	
Primer56	AGGCAAGAGCAATCAGGATC	
Primers used for examining double crossover events of *scrK* gene		
Primer61	TCAACTGAATGGGTAACAGT	
Primer64	TGGTCAATCGTAAGACTTCC	
Primers used for examining double crossover events of *hasE* gene		
Primer467	ATGTGGCAACCATTCTCACA	
Primer470	CTTCTGAGCCTTCTAAACC	
Primers used for examining double crossover events of *pfk* gene		
Primer37	TTAACAATTTGGAAGGGCTG	
Primer38	ATTGCTTTACGAACAACTGC	
Primers used for construction of the vectors for *scrA* overexpression		
Primer493	CATGCATGCCTGCAGCATATGTCAAACGCTTGACA	*Pst*I
Primer494	CGGAATTCGTCGACAGCAATGCACAAAGTATAGG	*Eco*RI
Primers used for construction of the vectors for *scrB* overexpression		
Primer495	CATGCATGCCTGCAGCATCTGAGGGACTTATCAGG	*Sph*I
Primer496	CGGAATTCGTCGACTCCTGCTGGCTAGCTCAACA	*Sal*I
